# Attitudes and perceptions about ageism among nursing students: a scoping review

**DOI:** 10.1590/1518-8345.6851.4116

**Published:** 2024-03-15

**Authors:** Jack Roberto Silva Fhon, Natalia Alves, Alexandre Pereira dos Santos, Alice Regina Felipe Silva Djinan, Anaclara Viggiano Laurenti, Eveline Fontes Costa Lima

**Affiliations:** Universidade de São Paulo, Escola de Enfermagem, São Paulo, SP, Brazil.; Scholarship holder at the Programa Unificado de Bolsas da Universidade de São Paulo, São Paulo, SP, Brazil.; Scholarship holder at the Coordenação de Aperfeiçoamento de Pessoal de Nível Superior (CAPES), Brazil.; Scholarship holder at the Conselho Nacional de Desenvolvimento Científico e Tecnológico (CNPq), Brazil.

**Keywords:** Ageism, Nursing Students, Review, Aged, Attitude, Perception, Ageismo, Estudiantes de Enfermería, Revisión, Anciano, Actitud, Percepción, Etarismo, Estudantes de Enfermagem, Revisão, Idoso, Atitude, Percepção

## Abstract

**Objective:**

to map scientific knowledge on nursing students’ attitudes and perceptions regarding ageism.

**Method:**

scoping review according to the recommendations of the Joanna Briggs Institute. The study question was: What are scientific evidence available on the attitudes and perceptions of nursing students regarding ageism? The search was conducted in 12 databases using the Rayyan application and the Preferred Reporting Items for Systematic reviews and Meta-Analyses extension for Scoping Reviews. The studies were selected by two reviewers using a data extraction tool.

**Results:**

of the 4,595 files, 46 that were published between 1984 and 2022 were selected, and the quantitative method was the most used. The most commonly used instrument was the Kogan’s Attitudes Toward Old People Scale. Positive, negative, mixed, neutral, and inconclusive attitudes and perceptions were identified.

**Conclusion:**

attitudes and perceptions about ageism are diverse and not conclusive. Future intervention studies are recommended to detect changes in the behavior of nursing students in the face of ageism.

## Resumen

**Objetivo:**

mapear el conocimiento científico sobre las actitudes y percepciones de los estudiantes de enfermería respecto al edadismo.

**Método:**

La pregunta del estudio fue: ¿Qué evidencia científica está disponible sobre las actitudes y percepciones de los estudiantes de enfermería respecto al edadismo? La búsqueda se realizó en 12 bases de datos utilizando la aplicación Rayyan y la *Preferred Reporting Items for Systematic reviews and Meta-Analyses extension for Scoping Reviews* . Los estudios fueron seleccionados por dos revisores mediante una herramienta de extracción de datos.

**Resultados:**

de los 4.595 artículos, se seleccionaron 46 publicados entre 1984 y 2022, siendo el método cuantitativo el más utilizado. La herramienta más utilizada fue el *Kogan’s Attitudes Toward Old People Scale* . Se identificaron actitudes y percepciones positivas, negativas, mixtas, neutras y no concluyentes.

**Conclusión:**

las actitudes y percepciones sobre la discriminación por edad son diversas y no concluyentes. Se recomiendan futuros estudios de intervención para detectar cambios en el comportamiento de los estudiantes de enfermería ante el edadismo.

## Resumo

**Objetivo:**

mapear o conhecimento científico sobre as atitudes e percepções dos estudantes de enfermagem em relação ao idadismo.

**Método:**

revisão de escopo segundo as recomendações do *Joanna Briggs Institute* . A pergunta de estudo foi: Quais são as evidências científicas disponíveis sobre as atitudes e percepções dos estudantes de enfermagem no que diz respeito ao idadismo? A busca foi realizada em 12 bases de dados utilizando o aplicativo Rayyan e o *Preferred Reporting Items for Systematic reviews and Meta-Analyses extension for Scoping Reviews* . Realizou-se a seleção dos estudos por dois revisores, com uso de um instrumento para a extração dos dados.

**Resultados:**

dos 4.595 arquivos, selecionaram-se 46 publicados entre 1984 e 2022, sendo que a maioria utilizou o método quantitativo. O instrumento mais utilizado foi o *Kogan’s Attitudes Toward Old People Scale* e os estudos mostraram que os estudantes apresentaram atitudes e percepções positivas, negativas, mistas, neutras e não conclusivas.

**Conclusão:**

as atitudes e percepções sobre idadismo são diversificadas e não conclusivas. Recomenda-se estudos futuros de intervenção para detecção de mudanças de comportamento dos estudantes de enfermagem frente ao idadismo.

**Highlights:**

**(1)** Attitudes and perceptions about ageism are diverse and not conclusive.

**(2)** Importance of discussing ageism in the training of nursing students.

**(3)** Intervention studies to identify paradigm shifts in nursing students.

## Introduction

 Aging is considered a physiological process that occurs throughout life; it is a natural process with morphological, functional, and biochemical changes, significant modifications to biological and psychological aspects that may compromise the autonomy of the elderly person, with greater susceptibility and vulnerability to the appearance of chronic non-communicable diseases ^(^ ^1^ ^-^ ^2^ ^)^ , which creates a scenario for the most diverse health concerns in this population ^(^ ^3^ ^)^ . 

 Understanding aging is associated with understanding the changes in order to create strategies that mitigate the effects of senescence. By ensuring social rights such as health, work, social assistance, education, culture, sport, transportation, autonomy, integration, and effective participation in society in the formulation and implementation of specific public and social policies, it qualitatively guarantees a well-succeeded old age ^(^ ^4^ ^)^ . 

 With the changes, it is noticeable that misinformation about the main challenges of the ageing population and the health of the elderly in the social context reinforces stigmas, which are responsible for building a society uncapable of producing changes in its social paradigm. In turn, social depreciation, characteristic of stereotypes, contributes to the emergence of prejudice and discrimination ^(^ ^5^ ^)^ . 

 In light of this, the American Gerontological Society has called ageism the prejudice committed against older people based on their age group, which directly impacts social needs, especially regarding health, and the rights of older people to age with dignity and quality of life ^(^ ^6^ ^)^ . In this context, age prejudice arises from categorizing and segregating people from different age groups, as ageism takes on discriminatory forms, leading to the weakening of care, work, political and personal relationships, as well as affecting perceptions and communication between individuals ^(^ ^7^ ^)^ . 

 This situation, when inserted into training environments for health professionals, as well as nursing, allows the manifestation of ageism, individually and institutionally, which can hinder the care of the elderly and the struggle against stereotypes, which affect the rights and integrity of this population ^(^ ^5^ ^)^ . 

 In a study carried out in Australia, known as ROPE (Relating to Older People Evaluation), the authors, aiming to evaluate ageing-related behaviours in nursing students, identified stigmatized and negative attitudes in 87.5% of the participants which occasionally hampers specialized care and interpersonal relationships concerning older people ^(^ ^8^ ^)^ . 

 In an attempt to understand nursing students’ perceptions of social discrimination against the elderly, especially among nursing students, a Brazilian study, based on questionnaires, recognized discriminatory and naturalized attitudes towards this population, according to ageist practices among university students ^(^ ^9^ ^)^ . 

With demographic and epidemiological shifts and the increase of the elderly population, newly graduated health professionals, including nurses, must be prepared to care for this population. In this sense, identifying ageist attitudes and perceptions among nursing students is important in order to combat misinformation and prejudice against the elderly during their training and the process of care and the biopsychosocial well-being of older people.

In a preliminary review of the International Prospective Register of Systematic Reviews (PROSPERO), Online System for Search and Analysis of Medical Literature (MEDLINE), the Cochrane Database of Systematic Reviews, the Joanna Briggs Institute (JBI) Evidence Synthesis and the Open Science Framework (OSF), no existing systematic or scoping review was identified. The aim of this scoping review was therefore to map scientific knowledge on nursing students’ attitudes and perceptions regarding ageism.

## Methods

### Type of study

 This study comprises a scoping review, developed according to JBI recommendations, which makes it possible to map the main concepts, clarify areas of research and identify knowledge gaps ^(^ ^10^ ^)^ . The first search in the different databases was carried out on January 21, 2022, and updated on October 5, 2022. The review protocol is registered in the OSF Registries at the link https://doi.org/10.17605/OSF.IO/Q5UF6 . 

### Data collection

The study question was developed using the acronym PCC according to the JBI methodology. The population (P) was considered to be nursing students (aged 18 years or older); the concept of interest (C) was attitude and perception about ageism; and the context (C) considered was nursing education institutions, formulating the following study question: What are scientific evidence available on the attitudes and perceptions of nursing students regarding ageism?

 For this scoping review, studies on the attitudes and perceptions of nursing students regarding ageism were considered. Thus, ageism consists of thoughts and attitudes directed towards people based on their age, and can be observed in institutional, interpersonal, and self-directed ways ^(^ ^7^ ^)^ . This review looked at studies carried out in universities, colleges and/or technical teaching institutions and nursing assistants, which have been developed worldwide. 

The search for published research was carried out in the following databases: MEDLINE (access via PubMed); Cumulative Index to Nursing and Allied Health Literature (CINAHL); Excerpta Medica Database (EMBASE); Scopus, Web of Science; and Latin American and Caribbean Health Sciences Literature (LILACS).

The search for unpublished studies, known as gray literature, came from: Google Scholar; the Brazilian Digital Library of Theses and Dissertations of the Coordination for the Improvement of Higher Education Personnel (CAPES); Networked Digital Library of Theses and Dissertations (NDLTD); Elton Bryson Stephens Company (EBSCO); Open Dissertations, Digital Access to Research Theses - Europe (DART-E); and the American Chemical Society Guide to Scholarly Communication.

 The search for studies followed three stages: 1) initial search in MEDLINE and Scopus to identify studies on the subject and select the words and indexing terms contained in these publications; 2) use of the keywords and terms identified for the search in databases; and 3) identification and selection of the articles contained in the reference lists of the sources used. The search strategies for the various databases are described in Figure 1 . 

**Figure 1:** - Search strategies and databases used in the literature review. São Paulo, 2022

Databases	Search strategies
VHL*	((“Estudantes de Enfermagem” OR “Students, Nursing” OR “Estudiantes de Enfermería”) AND ((“Atitude do Pessoal de Saúde” OR “Attitude of Health Personnel” OR “Actitud del Personal de Salud”) OR (Percepção OR Percepções OR Perception OR Perceptions))) AND ((Envelhecimento OR Aging OR Envejecimiento) OR (Ageismo OR Ageism OR Ageísmo))
MEDLINE ^†^	((“Students, Nursing” OR “Nursing Student” OR “Nursing Students”) AND (“Attitude of Health Personnel” OR Perception OR Perceptions)) AND (Aging OR Ageism)
CINAHL ^‡^	((“Students, Nursing” OR “Nursing Student” OR “Nursing Students”) AND (“Attitude of Health Personnel” OR Perception OR Perceptions)) AND (Aging OR Ageism)
EMBASE ^§^	‘Nursing student’ AND (‘health personnel attitude’ OR perception) AND (aging OR ageism)
Web of Science	((“Nursing Student” OR “Nursing Students”) AND (“Attitude of Health Personnel” OR Perception OR Perceptions)) AND (Aging OR Ageism)
Scopus	((“Nursing Student” OR “Nursing Students”) AND (“Attitude of Health Personnel” OR Perception OR Perceptions)) AND (Aging OR Ageism)
CAPES Brazilian Digital Library of Theses and Dissertations ^ǀǀ^	“Estudantes de enfermagem” AND Envelhecimento
Google Scholar	(“Nursing students” AND Perception) AND Ageism
EBSCO ^¶^	Open Dissertations “nursing students” AND Ageism
DART-E ^**^	“Nursing students” AND Ageism “Nursing students” AND Aging
IBICT Digital Library of Theses and Dissertations ^††^	“Estudantes de Enfermagem” AND Ageism

* VHL = Virtual Health Library;

† MEDLINE = Medical Literature Analysis and Retrieval System Online;

‡ CINAHL = Cumulative Index to Nursing and Allied Health Literature;

§ EMBASE = Excerpta Medica Database;

ǀǀ CAPES = Coordination for the Improvement of Higher Education Personnel;

¶ EBSCO = Elton Bryson Stephens Company;

** DART-Europe = Digital Access to Research Theses – Europe;

†† IBICT = Brazilian Institute of Science and Technology Information

 The search results have been reported in full in the final scoping review and presented in the Preferred Reporting Items for Systematic reviews and Meta-Analyses extension for Scoping Reviews (PRISMA-ScR) ^(^ ^11^ ^)^ . 

### Selection criteria

The review covered experimental and quasi-experimental studies involving randomized and non-randomized controlled trials, before-and-after studies and interrupted time series studies; analytical observational studies such as prospective and retrospective cohort studies; case-control studies and cross-sectional analytical studies; descriptive observational studies such as case series; individual case reports and descriptive cross-sectional studies; and studies with qualitative methodology and systematic reviews, which met the inclusion criteria.

Furthermore, texts from international and national bodies on the subject were analyzed. The inclusion criteria were publications with different methodologies, with no cut-off dates or language of publication. Publications that did not fit the study’s question and objective or that did not contain information on the study topic were excluded.

 After searching the databases, all the records identified were grouped together and loaded into the Rayyan application (Intelligent Systematic Review) ^(^ ^12^ ^)^ and in the first step duplicates were removed. Then, the titles and abstracts were read and selected by two independent reviewers for evaluation, applying the inclusion and exclusion criteria. Potentially relevant articles were retrieved in full and data extraction was carried out using a form created and developed by the authors and based on the form suggested by the JBI manual. 

The full text of the selected citations was assessed in detail against the inclusion criteria by two independent reviewers. The reasons for excluding full-text articles that did not meet the inclusion criteria were recorded and reported in the scoping review. Disagreements that arose between the reviewers at each stage of the selection process were resolved through discussion or with a third reviewer.

### Data collection tool

The data extracted included title, authors, year of publication, country of the study, objectives of the study, method used by the authors, instruments used to identify the phenomenon of study, demographic data such as gender and age of participants, attitudes, and perceptions of nursing students towards ageism.

### Data processing and analysis

Based on the data extracted, a descriptive analysis was carried out and tables were built with data from the publications, information on the sample, gender and age of the participants, instruments used and identification of the attitudes and perceptions of the participants.

## Results

**Figure 2:**
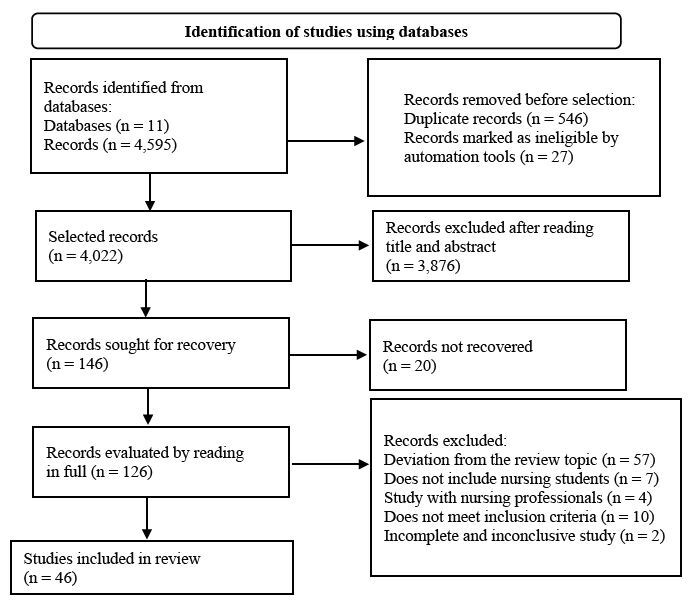
- PRISMA-ScR ^*^ flowchart used to identify and select studies. São Paulo, 2022

^*^ PRISMA-ScR = Preferred Reporting Items for Systematic reviews and Meta-Analyses extension for Scoping Reviews

 When searching the databases, 4,595 publications were identified and 546 duplicates were eliminated, leaving 4,022 files to be selected. In the first selection, by reading the titles and abstracts and applying the inclusion and exclusion criteria with two independent reviewers, 3,876 were eliminated, leaving 146 files. The material was then read, and the inclusion and exclusion criteria were applied once again, leaving a final sample of 46 publications ( Figure 2 ). 

The search methods most used by the authors were: quantitative (34; 73.9%), qualitative (5; 10.8%), review (3; 6.6%), mixed (3; 6.6%), and randomized clinical trial (1; 2.1%). The main language of publication was English (34; 73.9%), followed by Portuguese (4; 8.7%) and Spanish (4; 8.7%), while there were four articles in different languages, published in Turkish (2; 4.3%), Croatian (1; 2.2%), and Thai (1; 2.2%).

With regard to the description of the studies, it was identified that the years of publication were between 1984 and 2022, with the largest number of published studies being carried out in 2022 (11; 23.9%), followed by 2021 (7; 15.2%), 2016, and 2015 (4; 8.6%), respectively.

The countries that did the most research on the subject were the United States (9; 19.5%), China (7; 15.2%), Turkey (6; 13.0%) and Australia (4; 8.7%).

 The studies identified 11,121 participants, 8,035 of whom were female. Regarding the most commonly used instrument in the different studies, it was found that 15 (32.6%) used the Kogan’s Attitudes Toward Old People Scale (KAOP) ( Figure 3 ). 

**Figure 3:** - Identification of the article by author, title, objective, type of study, and country (n * =46). São Paulo, 2022

Author	Objective	Type of study	Sample / population	N* men/ women	Age	Instrument	Country of study	Publication language
America								
Enríquez-Reyna, et al. ^(^ ^13^ ^)^	Assessing attitudes and perceptions, by gender, of female and male aging among undergraduate nursing students in Zacatecas, Mexico.	Quantitative and cross-sectional	262	77 (29.4%) / 185 (70.6%)	Mean 21.4 years DP ^†^ =2.87 years	Kogan’s Attitudes Toward Old People Scale	Mexico	Spanish
Leitón-Espinoza ^(^ ^14^ ^)^	To determine the relationship between sociodemographic factors and negative stereotypes of old age among nursing students at the Nursing School of the National University of Trujillo.	Quantitative and cross-sectional	236	14 (5.9%) / 222 (94.1%)	Predominance of 18-29 year olds (99.6%)	Negative Stereotypes of Age	Peru	Spanish
Brown; Wang ^(^ ^15^ ^)^	To explore nursing students’ perceptions of the elderly and evaluate learning activities aimed at reducing ageism myths.	Mixed	102	7 (7%) / 95 (93%)		Fraboni Scale of Ageism	United States	English
Chance, et al. ^(^ ^16^ ^)^	To learn more about the attitudes of nursing students towards the elderly in the United States and Costa Rica.	Quantitative and cross-sectional	269	-	From 19 years old	Kogan Old Persons Scale	United States	English
Dahlke, et al. ^(^ ^17^ ^)^	To generate evidence on the effectiveness of the three e-learning activities on nursing students’ perceptions of the elderly.	Quasi- experimental	640	-		Burbank’s Perceptions About Older People; The Ambivalent Ageism Scale	Canada	English
Hovey, et al. ^(^ ^18^ ^)^	To analyze empirical studies from the United States and Canada to understand how nursing education affects nursing students’ attitudes towards older people.	Integrative literature review					United States and Canada	English
Duran-Badillo, et al. ^(^ ^19^ ^)^	To identify and describe the stereotypes nursing students have about the elderly.	Quantitative and cross-sectional	68		Mean 22 years old	Negative Stereotypes of Old Age Questionnaire	Mexico	Spanish
Alexandre, et al. ^(^ ^20^ ^)^	To understand nursing students’ perceptions of the elderly and old age.	Qualitative	30	3 (8.55%) / 27 (91.45%)	Mean 24 years old	Self-reported questionnaire	Brazil	Portuguese
Lima; Oliveira ^(^ ^21^ ^)^	To understand how nursing students’ prejudice towards the elderly influences their nursing practice.	Systematic review					Brazil	Portuguese
Shortreed ^(^ ^22^ ^)^	To compare the attitudes of second and final year undergraduate nursing students towards the elderly. The intent of this study was to help determine whether ageist attitudes are observed prior to clinical experiences and also how students’ attitudes change in the final year after completing the bulk of the nursing curriculum.	Quantitative and cross-sectional.	38	3	(7.9%) / 35 (92.1%)		Kogan’s Attitude Toward Old People Scale	United States	English
Mattos, et al. ^(^ ^23^ ^)^	To explore the knowledge and attitudes of undergraduate nursing students about the elderly. Specific research questions included.	Mixed	132	12 (9.3%) / 120 (90.7%)	Predominance of 21 year olds	The Facts of Aging – Form 2; The Geriatric Attitudes Scale	United States	English
Lee ^(^ ^24^ ^)^	To identify attitudes and prejudices towards ageing among Asian and White students and to identify factors affecting attitudes towards ageing.	Quantitative and cross-sectional	308	39 (12.7%) / 268 (87.3%)	79.3% under 25; 10.5% between 25-30 and 10.2% 30 and over	Kogan’s Attitude Toward Old People Scale; Aging Quiz electronically	United States	English
Friday ^(^ ^25^ ^)^	To examine the impact of a four-week educational intervention with gerontological content on nursing students’ perception of the elderly.	Qualitative	8	2 (25%) / 6 (75%)	75% between 20 and 39 years old	Kogan’s Attitudes Toward Old People scale	United States	English
Haight; Christ; Dias ^(^ ^26^ ^)^	To examine the impact of selected learning experiences of undergraduate nursing students’ attitudes towards older people.	Quantitative and cross-sectional	57	5 (8.8%) / 52 (91.2%)	70% between 20 and 40 years old	Kogan’s Attitudes Toward Old People scale	United States	English
Melanson; Downe-Wamboldt ^(^ ^27^ ^)^	To determine the relationship between the selected independent variables and the attitudes of undergraduate nursing students towards the elderly.	Quantitative and cross-sectional	122	-		Opinions About People	Canada	English
Goebel ^(^ ^28^ ^)^	To determine whether age stereotypes held by nursing students reflect cultural attitudes before they become practicing professionals.	Quantitative and cross-sectional	72	- / 72 (100%)		Kogan’s Attitudes Toward Old People Scale;	Attitudes toward old people scale	United States	English
Europe								
Vincek ^(^ ^29^ ^)^	To verify whether there are differences in knowledge about the elderly among students from different periods of nursing school. The aim was to determine whether there are differences between students’ attitudes and perceptions of the elderly, based on their knowledge during their undergraduate studies.	Quantitative and cross-sectional	102	22 (22%) / 80 (78%)	Aged between 19 and 33. 31.3% were 21 years old	Semantic Differential of Attitudes Towards the Elderly	Croatia	Croatian
Turan; Polat; Çiftçi ^(^ ^30^ ^)^	To find out about the attitudes of nurses who are students on the geriatric nursing course and those who are not on the course at two different universities towards old age and ageing.	Quantitative and cross-sectional	181	35 (19.3%) / 146 (80.7%)	Mean 21.64 years old	Attitude Scale Toward Aging and Elderliness	Turkey	Turkish
Castellano-Rioja, et al. ^(^ ^31^ ^)^	To measure attitudes towards the elderly in health professionals, as there is a need to implement interventions to improve attitudes towards the elderly from the training of nursing students.	Quantitative and cross-sectional	97	-	Mean 22.5 years old	Kogan’s Attitudes Toward Old People Scale	Spain	English
Hançerlioğlu; Toygar; Theofanidis ^(^ ^32^ ^)^	To explore the attitudes of nursing students in Turkey towards ageing and old age and to determine differences according to the year of study.	Quantitative and cross-sectional	287	28 (9.5%) / 259 (90.5%)	Mean 21.4 ± 1.2 years old	Attitudes Toward Ageing and Elderliness Scale	Turkey	English
López-Hernández, et al. ^(^ ^33^ ^)^	To describe attitudes towards the elderly in a sample of nursing students and to analyze the potential factors that influence these attitudes.	Quantitative and cross-sectional	377	91 (24.1%) / 286 (75.9%)	Mean 22.23 years old	Kogan’s Attitudes Toward Old People Scale	Spain	English
Serin; Tülüce ^(^ ^34^ ^)^	To determine the attitudes and empathic tendencies of nursing students regarding discrimination against the elderly.	Quantitative and cross-sectional	229	51 (22.3%) / 178 (77.7%)	Mean 20.78 ± 1.54 years old	The Ageism Attitude Scale; The Empathic Tendency Scale	Turkey	English
Sinan; Bilgili; Mutlu ^(^ ^35^ ^)^	To study and indicate the relationships and attitudes identified in nursing students regarding the treatment of the elderly and older people, as well as concepts of old age.	Quantitative and cross-sectional	543	61 (11.2%) / 482 (88.8%)	Mean 22.00 ± 1.20 years old	Attitudes and Knowledge Towards Older People; Attitudes of Healthcare Workers; Attitudes of Turkish Nursing Students Related to Ageism	Turkey	English
Darling, et al. ^(^ ^36^ ^)^	Assessing the motives and perceptions of nursing students about a career in gerontology.	Quantitative and cross-sectional	468	61 (13%) / 407 (87%)	Age between 17 and 30, average of 20.6 ± 1.9 years old	Kogan’s Attitude Toward Old People Scale	Turkey	English
Ridgway ^(^ ^37^ ^)^	To visually and critically explore the perceptions of ageing held by undergraduate nursing students at a university in the north of England.	Quantitative and longitudinal	307	28 (9%) / 279 (91%)	52% between 17 and 21; 25% between 22 and 29; 15% between 30 and 39; and 8% above 40 years old	Kogan’s Attitude Toward Old People Scale	England	English
Sarabia; Castanedo ^(^ ^38^ ^)^	To explore the modification of stereotypes and myths about old age in third-year nursing students before and after learning the subject Gerontological Nursing.	Quantitative and cross-sectional	76	9 (11.6%) / 67 (88.4%)	Mean 20.37 years old	The Negative Stereotypes Questionnaire about Aging	Spain	Spanish
Fontes ^(^ ^39^ ^)^	To understand nursing students’ perceptions of social discrimination against the elderly.	Quantitative and cross-sectional	78	23 (29.5%) / 55 (70.5%)	Mean 23.31 years old	Old Age Image Scale	Portugal	Portuguese
Adibelli; Türkoğlu; Kiliç ^(^ ^40^ ^)^	Determine nursing students’ views on ageing and their attitudes towards the elderly.	Quantitative and cross-sectional	308	88 (28.6%) / 220 (71.4%)	Most between 21 and 24 years old	Kogan’s Attitude Toward Old People Scale	Turkey	Turkish
Magalhães, et al. ^(^ ^41^ ^)^	To identify the most prevalent representational contents that first-year nursing students have constructed about ageing and old age, gerontological nursing and geriatric nursing, prior to learning contents in this area.	Qualitative	42	7 (16.67%) / 35 (83.33%)	Mean 19.55 years old	Self-reported questionnaire	Portugal	Portuguese
Usta, et al. ^(^ ^42^ ^)^	To examine the attitudes of 145 Turkish nursing students studying about ageism and the factors that affect their view of this problem.	Quantitative and cross-sectional	145	17 (11.7%) / 128 (83.3%)	Mean 20.10 years old	The Ageism Attitude Scale	Ireland	English
Karlin, et al. ^(^ ^43^ ^)^	Comparing age discrimination in psychology and nursing students.	Quantitative and cross-sectional	81	12 (14.8%) / 69 (85.2%)	Mean 31.3 years old ranging from 20-55 years	Polizzi’s Refined Scale for Elder Adults	England	English
Reed; Beall; Baumhover ^(^ ^44^ ^)^	Examining knowledge about ageing and attitudes towards the elderly among master’s students in social work and nursing.	Quantitative and longitudinal	27	-	Ranging from 21-50, mean 33.9 years old	Old People Scale	UK	English
**Asia**								
Phisaiphanth; Vongtree; Chabuakam ^(^ ^45^ ^)^	To study the knowledge of caring for the elderly and to identify attitudes towards caring for the elderly among nursing students and the relationship between knowledge and attitudes towards caring for the elderly among nursing students at Boromarajonani Sapasithiprasong Nursing College in Ubon Ratchathan Province.	Quantitative and cross-sectional	138	-	Mean 20.14 SD ^†^ =1.02 years	The Palmore Facts on Aging Questionnaire; The Kogan’s Old People Scale Questionnaire	Thailand	Thai
Zhang, et al. ^(^ ^46^ ^)^	To explore the factors associated with attitudes towards the elderly among nursing students, to clarify the impact of empathy and end of life care on the attitude of the elderly; and to serve as a basis for the follow-up of education courses related to the care of the elderly and training of caregivers of the elderly talents.	Quantitative and cross-sectional	371	41 (11.05%) / 330 (88.95%)		The End-of-life Attitudes Scale; Kogan Attitudes Scale for the Elderly	China	English
Fu, et al. ^(^ ^47^ ^)^	Exploring attitudes and factors towards the elderly among undergraduate nursing students at national colleges in the Heilongjian province.	Quantitative and cross-sectional	978	155 (15.85%) / 823 (84.15%)		Kogan’s Attitudes Toward Old People Scale	China	English
Cheng ^(^ ^48^ ^)^	To assess students’ preparation, perspectives, attitudes, and knowledge about ageing, as well as their intention to work in the gerontological field.	Quantitative and cross-sectional	139	34 (24.5%) / 105 (75.5%)	82.7% between 19-23 years old	The Willingness to Care for Older People	China	English
Cheng, et al. ^(^ ^49^ ^)^	To investigate the effectiveness of a Senior Simulation Program (SSSP). The SSSP ^‡^ , which focused on mimicking the physiological experiences of an 80-year-old person, was hypothesized to increase the user’s positive attitude towards elderly care.	Randomized clinical trial	139	34 (24.5%) / 105 (75.5%)	87% were 23 years or younger; 13% older than 23 years old	Kogan’s Attitudes Toward Old People Scale; The Willingness to Care for Older People	China	English
Hsu; Ling; Lui ^(^ ^50^ ^)^	To explore the information and attitudes of nursing students towards the elderly and to examine the presence of relationships between these factors and the teaching of gerontological nursing in Macau, a special administrative region of China.	Quantitative and cross-sectional	484	56 (14.9%) / 321 (85.1%)	Mean 21.3 years old	Kogan’s Attitudes Toward Old People Scale	China	English
Zverev ^(^ ^51^ ^)^	Exploring the attitudes of medical and nursing students in Malawi towards the elderly.	Quantitative and cross-sectional	151	28 (18.5%) / 123 (81.5%)	Mean 21 years old	Kogan’s Attitudes Toward Old People Scale	China	English
Jo; An ^(^ ^52^ ^)^	Exploring the perception of ageing among undergraduate nursing students.	Qualitative	102	3 (2.9%) / 99 (97.1%)	Most were around 19 years old	Self-reported questionnaire	South Korea	English
Oceania								
Dahlke, et al. ^(^ ^53^ ^)^	Gain an understanding of students’ perceptions of working with older people.	Quantitative and cross-sectional	370	33 (8.9%) / 337 (91.1%)	50% were 21 years or older	Burbank’s Perceptions of Caring for Older People’s scale	Australia	English
Frost; Ranse; Grealish ^(^ ^8^ ^)^	Describe the prevalence of ageist behavior in first-year undergraduate nursing students.	Quantitative and cross-sectional	180	31 (15.1%) / 149 (82.7%)	Mean 24 years old	Relating to Older People Evaluation	Australia	English
Neville; Dickie ^(^ ^54^ ^)^	To assess undergraduate nurses’ attitudes and perspectives towards older people and perceptions of working with older people.	Literature review					Australia	English
Moyle ^(^ ^55^ ^)^	To identify nursing students’ views of the elderly in order to provide insights into how these perceptions can influence students’ choice of workplace and the care they may provide to the elderly.	Quantitative and longitudinal	103	12 (11.7%) / 91 (88.3%)	Mean 28 years old	Self-reported questionnaire	Australia	English
Africa								
Attafua, et al. ^(^ ^56^ ^)^	Exploring students’ perceptions of ageing and their attitudes towards caring for the elderly.	Qualitative	30	15 (50%) / 15 (50%)	Mean 22.30 years old	Self-reported questionnaire	Ghana	English
Multicentric								
Cheng, et al. ^(^ ^57^ ^)^	To examine the willingness to work with older people and associated factors among nursing students from nine countries (or regions).	Quantitative and cross-sectional	2244	549 (24.5%) / 1695 (74.5%)	Mean 20.56 SD ^†^ = 2.41 years old	Attitude Toward Aging; Older Person Care Perception	China, Palestine, Saudi Arabia, Chile, India, Philippines, Egypt, and Greece.	English

* n = Number;

† SD = Standard Deviation;

‡ SSSP = Senior Simulation Program

 Among the findings of the articles, 23.5% showed positive attitudes and perceptions and 19.6% negative attitudes and perceptions ( Figure 4 ). 

 Moreover, the results identified mixed attitudes and perceptions (9; 19.6%), neutral (2; 4.3%) and inconclusive (3; 6.5%) ( Figure 5 ). 

**Figure 4:** - Positive and negative attitudes and perceptions about ageism among nursing students. São Paulo, 2022

Article	Positive attitudes and perceptions
Enríquez-Reyna, et al. ^(^ ^13^ ^)^	The majority of participants had a positive attitude towards the elderly.
Brown; Wang ^(^ ^15^ ^)^	There was no statistically significant difference in the perception of pre- and post-intervention students, however, in general, positive words were used to describe the elderly, such as wise, kind, and gentle.
Chance. et al. ^(^ ^16^ ^)^	It was found that pre-licensure nursing students living in the USA* reported positive attitudes towards the elderly.
Dahlke, et al. ^(^ ^17^ ^)^	The sample’s average score on the instrument used indicated a slightly positive outlook towards older people.
Lima; Oliveira ^(^ ^21^ ^)^	There was a greater tendency for nursing students to have positive attitudes towards older people.
Shortreed ^(^ ^22^ ^)^	Participants entered and left the nursing course with very positive attitudes towards older people.
Mattos, et al. ^(^ ^23^ ^)^	Nursing students had a positive attitude towards the elderly according to the instrument used.
Haight; Christ; Dias ^(^ ^26^ ^)^	Different positive attitudes were attributed to nursing students.
López-Hernández, et al. ^(^ ^33^ ^)^	Attitudes towards the elderly among nursing students were positive, with women having a more positive attitude than men.
Darling, et al. ^(^ ^36^ ^)^	The scores of the nursing students indicated positive attitudes.
Ridgway ^(^ ^37^ ^)^	The majority of participants had moderately positive attitudes towards the elderly.
Fontes ^(^ ^39^ ^)^	The results showed that there is an overall positive view of ageism.
Karlin, et al. ^(^ ^43^ ^)^	Nursing students showed higher levels of efficacy when it came to working with the elderly population compared to psychology students. In addition, nursing students reported low levels of ageism.
Phisaipan; Wongtri; Chabuakham ^(^ ^45^ ^)^	The majority of nursing students said they had good knowledge about caring for the elderly, and also said they had positive attitudes towards ageing and the elderly.
Zhang, et al. ^(^ ^46^ ^)^	The students’ attitudes were positive, but slightly lower than the national average in China.
Fu, et al. ^(^ ^47^ ^)^	Students’ attitudes were positive, but the choice of gerontology/geriatrics as a first career option needs to increase.
Cheng ^(^ ^48^ ^)^	The majority of students scored highly on the scale used, indicating positive attitudes towards the elderly.
Cheng, et al. ^(^ ^49^ ^)^	A significant increase in positive attitudes and willingness to serve the elderly was found in both the control and intervention groups.
Hsu; Ling; Lui ^(^ ^50^ ^)^	The nursing students showed positive attitudes (high average KAOP ^†^ scores) towards the elderly.
Zverev ^(^ ^51^ ^)^	The majority of nursing students had positive attitudes towards the elderly.
Jo; An ^(^ ^52^ ^)^	The majority of students indicated positive engagement with the elderly.
Neville; Dickie ^(^ ^54^ ^)^	The attitudes, perspectives and perceptions of undergraduate nurses were positive.
Attafuah, et al. ^(^ ^56^ ^)^	The students see the elderly as their grandparents; therefore, they tend to treat this age group with respect.
Negative attitudes and perceptions	
Duran-Badillo, et al. ^(^ ^19^ ^)^	Analysis of the overall scale indicates that more than half of the students had negative stereotypes.
Alexandre, et al. ^(^ ^20^ ^)^	The students had negative ageist attitudes related to the meanings of old age, such as: experience, pejorative, stigma, changes, and special care, and for the elderly, dependence, fear and coping, patience, and stubbornness.
Mattos, et al. ^(^ ^23^ ^)^	Few students showed an interest in working in the field of elderly health and, compared to those who took the gerontology course, nursing students had negative perceptions.
Lee ^(^ ^24^ ^)^	The students had significantly more negative attitudes and anti-age prejudices towards the elderly.
Friday ^(^ ^25^ ^)^	The research showed that nursing students had negative attitudes.
Goebel ^(^ ^28^ ^)^	Nursing students not only endorsed negative characteristics as typical of the elderly, but also had significantly more negative attitudes towards the elderly.
Sarabia; Castanedo ^(^ ^38^ ^)^	High prevalence of negative stereotypes about old age among nursing students.
Magalhães, et al. ^(^ ^41^ ^)^	The results reveal that nursing students devalue the condition of the elderly.
Moyle ^(^ ^55^ ^)^	Nursing students are continuing society’s myths that the elderly are frail and show a decline in health.

* USA = United States of America;

† KAOP = Kogan’s Attitudes Toward Old People Scale

**Figure 5:** - Mixed, neutral, and inconclusive attitudes and perceptions about ageism among nursing students. São Paulo, 2022

Article	Mixed attitudes and perceptions
Frost; Ranse; Grealish ^(^ ^8^ ^)^	Both positive and negative attitudes towards the elderly were identified on the part of nursing students, given that the majority of students indicated that they would work in the care of the elderly in the future, while some reported engaging in some ageist behaviors.
Leitón-Espinoza ^(^ ^14^ ^)^	The predominant level of negative stereotyping was low, followed by high; the level of negative stereotyping regarding gender was not significant; in relation to age, in relation to year of course, it was not significant and in relation to contact with older people and negative stereotypes, there was no significant difference.
Castellano-Rioja, et al. ^(^ ^31^ ^)^	No changes were observed after the students completed the elderly care course. However, there was a significant change in participants’ attitudes after completing the clinical cycle, with an increase in positive attitudes, but negative attitudes did not decrease.
Hançerlioğlu; Toygar; Theofanidis ^(^ ^32^ ^)^	Differences were found regarding perceptions of social strain, difficulty coping with life, and negative images between students who do and do not care for the elderly.
Serin; Tülüce ^(^ ^34^ ^)^	More than half of the nursing students were willing to work with the elderly after graduating. However, students associated affection, weakness, illness, dependence, loneliness, and wisdom with the elderly.
Sinan; Bilgili; Mutlu ^(^ ^35^ ^)^	Nursing students’ perception of the elderly was positive; however, certain perspectives and behaviors regarding to caring for the elderly were negative.
Adibelli; Türkoğlu; Kiliç ^(^ ^40^ ^)^	It was found that nursing students’ views on ageing are mostly negative, and their attitudes towards the elderly are positive.
Neutral attitudes and perceptions	
Vincek ^(^ ^29^ ^)^	The vast majority of students took a neutral stance. The interviewees gave neutral answers to all the questions about attitudes towards people over 65 years old.
Turan; Polat; Çiftçi ^(^ ^30^ ^)^	There was no significant difference between the group that took the “Scale of Attitude Towards Ageing” course and the group that did not take the course.
Reed; Beall; Baumhover ^(^ ^44^ ^)^	Attitudes towards the elderly tend to be neutral rather than strongly positive or negative.
Inconclusive attitudes and perceptions	
Hovey, et al. ^(^ ^18^ ^)^	The research did not adequately respond to the results found, identifying that there are several gaps in the literature on the impacts of ageist attitudes among nursing students towards elderly care.
Melanson; Downe-Wamboldt ^(^ ^27^ ^)^	There was variation in attitude scores in four of the seven attitude dimensions, realistic harshness towards the elderly, anxiety about ageing, family responsibility and unfavorable stereotypes of the elderly, without quantifying and evaluating them conclusively.
Dahlke, et al. ^(^ ^53^ ^)^	Although ageist attitudes will be improved with the proposed activities, they do not identify them, making it inconclusive as to which attitudes were found in the students.

## Discussion

Attitudes and perceptions about ageism among nursing students were mapped and it was found that studies are being carried out on this subject in different countries, especially in developed countries with higher rates of elderly population. Furthermore, it was observed that the studies provide diverse results by identifying positive, negative, mixed, neutral, and inconclusive attitudes and perceptions.

 The United Nations report found that one in two people in the world have discriminatory attitudes that worsen the physical and mental health of older people and reduce their quality of life, costing billions of dollars every year ^(^ ^58^ ^)^ . In the United States, a study showed that discrimination - in the form of negative age stereotypes and self-perceptions - led to excessive annual costs of US$63 billion, equivalent to US$1 in every US$7 for all Americans over 60 years old for a year ^(^ ^58^ ^)^ . 

 Regarding the participants, the studies found that there was a predominance of women. This is due to the fact that nursing is a profession made up mostly of women ^(^ ^59^ ^)^ . Historically, care practices have been associated with the female gender since the dawn of civilization, in order to maintain survival, in which men dedicated themselves to providing food through hunting and fishing, and women were responsible for domestic work and caring for the sick ^(^ ^60^ ^)^ . 

 The studies found that the most commonly used instrument in this review was the KAOP, with adaptations for the reality of each country, as found in the literature. This instrument identifies intergenerational relationships in order to determine the meaning and intensity of respondents’ attitudes and perceptions towards older people in general; it is not restricted to health professionals alone and can be applied to different social groups ^(^ ^16^ ^)^ . It is therefore a suitable instrument for studying the relationship between attitudes and perceptions among students, allowing the degree of ageism to be assessed ^(^ ^61^ ^)^ . 

 The use of validated scales is very common in health research, since they are excellent instruments for measuring subjective issues of individuals or a certain group, as well as being able to measure behavioral patterns ^(^ ^62^ ^)^ . However, it is important to be aware of the cultural differences that a given scale may have in relation to its country of origin and country of application. The instrument should be adapted according to the country’s customs and habits, so that the results obtained are as close to reality as possible, as well as making it easier for the target audience to understand ^(^ ^62^ ^)^ . 

 The studies analyzed showed different attitudes and perceptions of nursing students about ageism. Among the positive attitudes found in the studies, we identified the description of the elderly as wise, kind, gentle and a high tendency to respect this age group ^(^ ^45^ ^,^ ^49^ ^,^ ^52^ ^)^ . There is a consensus that working with the elderly requires special training, a personal vocation, and a preference for working with this social group. It is imperative to include topics related to healthy ageing in the training of new nursing professionals, and not just topics related to pathologies and diseases associated with old age ^(^ ^63^ ^)^ . 

 With regard to negative attitudes, it was found that nursing students had negative stereotypes derived from stigma; the need for special care, their own dependence, a lack of patience for caring for the elderly, age-related prejudice, devaluing the elderly, and continuing society’s myths about the ageing process ^(^ ^19^ ^,^ ^24^ ^,^ ^38^ ^)^ . 

 The literature describes that among the negative stereotypes most frequently attributed to the elderly population are illness, incapacity, unproductivity, dependence, motor and cognitive decline, loss of strength or power, decadence, isolation, and social exclusion such as the inability to hire older candidates, among others ^(^ ^64^ ^-^ ^65^ ^)^ , due to the lack of an intergenerational relationship aimed at deconstructing old age stereotypes ^(^ ^66^ ^)^ , which is a challenge for society. 

 In this context, these erroneous generalizations attributed to elderly people can occur implicitly, through unintentional actions and thoughts activated automatically through previous beliefs, and negatively influence their self-image and experience of longevity ^(^ ^67^ ^)^ . 

 Society’s disdain for the elderly is partly due to a lack of knowledge about the ageing process, leading to the development of prejudices which are then translated into stereotypes and derogatory attitudes. It is therefore possible to reduce these negative perceptions of ageing through interventions that change them into positive perceptions of the ageing process and target attitudes in society. This should be done through changes in public policies aimed at the elderly population, as well as public campaigns and community education programs, thus reducing prejudice ^(^ ^68^ ^)^ . 

 Even so, in the studies that were included in the review, nursing students had mixed, neutral, and inconclusive attitudes and perceptions. A Polish study found that knowledge on ageing and contact with older people can significantly affect attitudes and behaviors towards ageing of the population ^(^ ^69^ ^)^ . 

 Nursing training should be based on the different national and international health policies. Despite older people’s many contributions to society and their great diversity, negative attitudes that are common in all societies are rarely challenged and can be disadvantageous for older women and older people with disabilities. In this sense, it is important to highlight actions against prejudice, such as supporting educational and intergenerational activities, campaigns against prejudice, and adopting or modifying legislation to prohibit discrimination, among other actions ^(^ ^70^ ^)^ . 

 Regular contact or cohabitation with the elderly is one of the main determinants in the expression of positive attitudes towards this population, underlining the need to integrate intergenerational contact as a differential element in the training of future health professionals. In light of the lack of such contact, it is possible to understand age prejudice in nursing students by structuring different strategies and learning that may reduce the attitudes and practices that reinforce such negative behaviors ^(^ ^8^ ^)^ . Moreover, training on ageing should be carried out throughout their professional training, as this will help to reduce existing stigmas which show more negative and neutral attitudes towards ageing ^(^ ^71^ ^)^ . 

The present review has some limitations that should be considered: as it was a scoping review, there was no quality assessment of the available evidence, and it was not possible to assess the implications for clinical practice. Moreover, most of the studies included were cross-sectional, which does not allow for long-term conclusions and statistical inferences to be made.

## Conclusion

Based on the analysis of the results of the 46 selected articles, it was observed that the studies bring diverse results by identifying positive, negative, mixed, neutral and inconclusive attitudes and perceptions. There was a predominance of positive attitudes from nursing students in the publications investigated. However, the studies analyzed also revealed the devaluation of the condition of the elderly by students. Attitudes, perceptions and age stereotypes, when held by healthcare professionals, can potentially affect their relationship with patients. Therefore, studies of this nature contribute to the advancement of knowledge by pointing out that the literature shows the difficulties of students in training in the face of ageism and that we have a challenge in the training of nursing students. The studies included in this review support future intervention studies to detect changes in the behavior of nursing students in the face of ageism in developed and developing countries.
